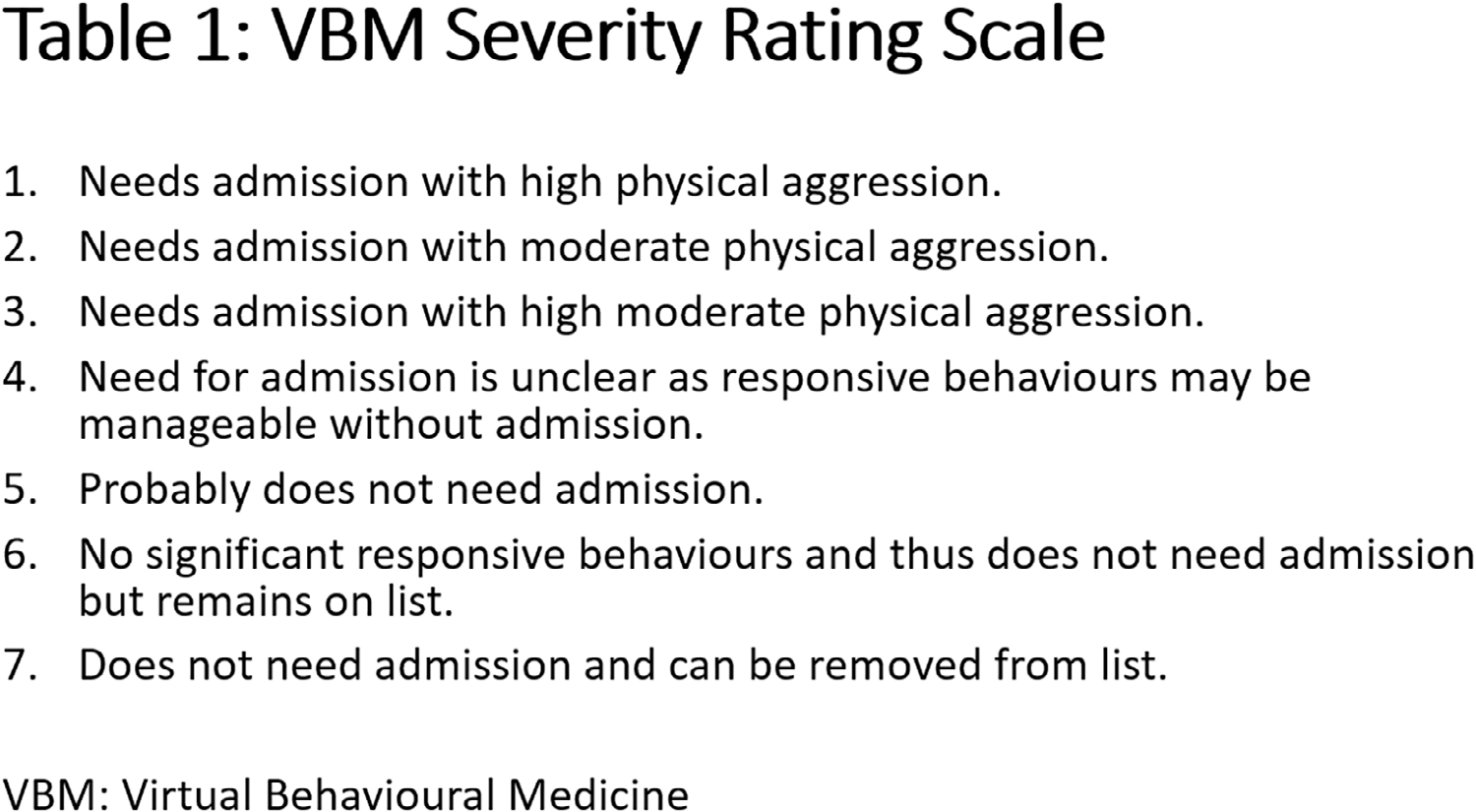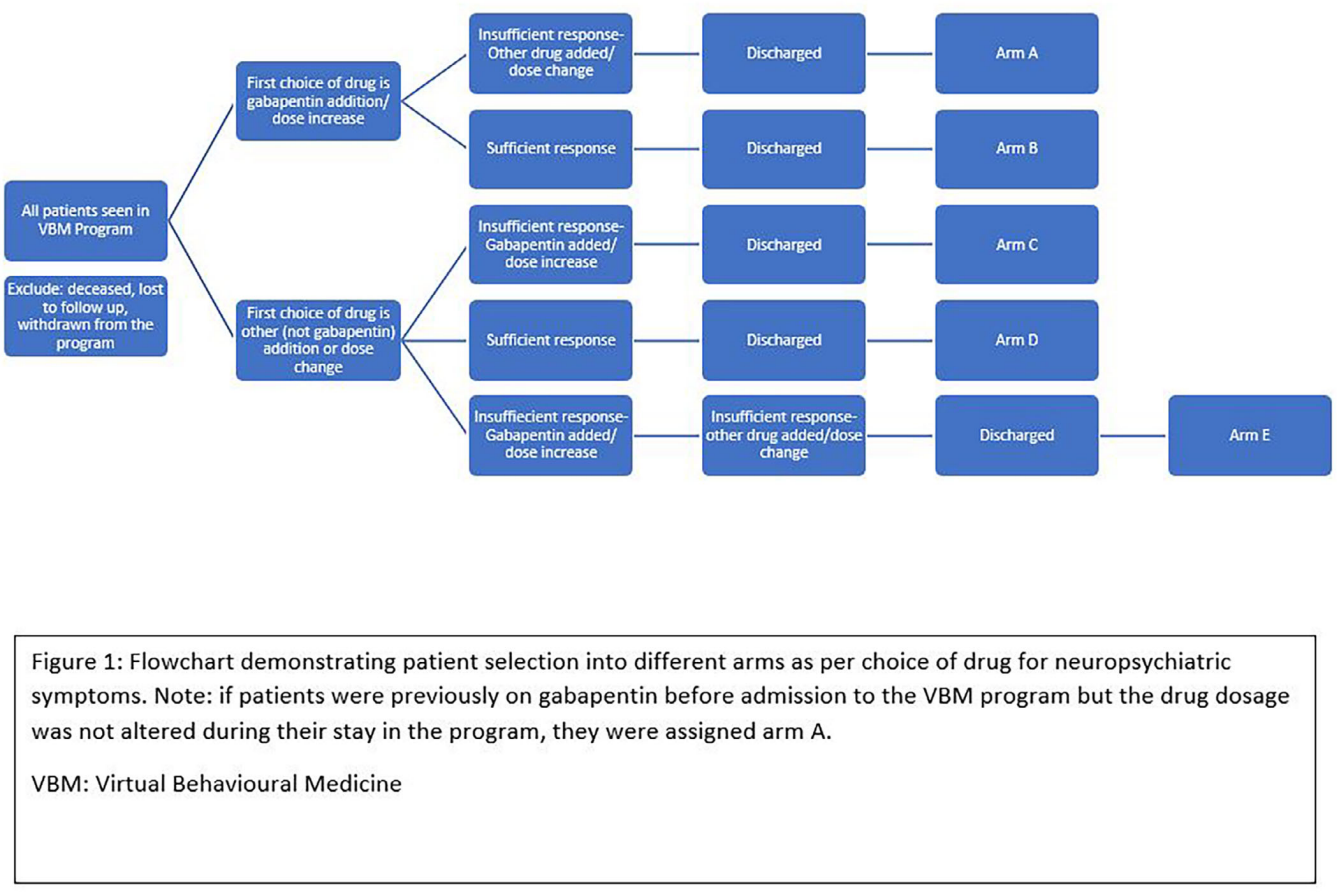# Gabapentin as an Effective Treatment of Neuropsychiatric Symptoms in Dementia

**DOI:** 10.1002/alz70861_108441

**Published:** 2025-12-23

**Authors:** Nodee Chowdhury, Carolyn Pavoni, Malcolm Binns, Sinjan Ghosh, Swayang Sudha Panda, Ericka Teleg, Morris Freedman

**Affiliations:** ^1^ Baycrest Health Sciences, Toronto, ON Canada; ^2^ University of Toronto, Toronto, ON Canada; ^3^ Dalla Lana School of Public Health, University of Toronto, Toronto, ON Canada; ^4^ Rotman Research Institute, Baycrest Academy for Research and Education, Toronto, ON Canada; ^5^ Mt. Sinai Hospital, Toronto, ON Canada; ^6^ Rotman Research Institute, Baycrest Health Sciences, Toronto, ON Canada

## Abstract

**Background:**

Neuropsychiatric symptoms (NPS) in dementia are challenging to manage. While Canadian Clinical Practice Guidelines recommend atypical antipsychotics, these often lead to extrapyramidal and gait‐related side effects. A 2018 algorithm by Davies et al. suggested that gabapentin, an anti‐epileptic drug, has limited evidence for managing agitation and aggression in dementia. This study aimed to evaluate the efficacy of gabapentin in treating NPS.

**Methods:**

Patients in the Virtual Behavioural Medicine (VBM) program from 2022‐ 2024 (*n* =250) were treated for NPS in advanced dementia. Demographic, clinical, and treatment data were reviewed. We used the Severity Rating Scale (SRS) to assess NPS severity, where lower scores indicate more severe symptoms (Table 1). Successfully discharged patients (SRS = 7) were categorized into five arms (A–E) (Figure 1). Arm B included patients treated solely with gabapentin, while arm C received at least one drug before successful treatment with gabapentin. Data were analyzed with LASSO and Cox proportional hazards regressions.

**Results:**

Gabapentin responders made up 23.6% (*n* =59, 95% CI: 18–29%) ‐ 48 in arm B and 11 in arm C. Arm B had the youngest median age (73 years, range 47–94), and a male predominance (1.67:1). At baseline, 73% in arm B exhibited physical aggression, 60% verbal aggression, and 52% agitation, while in arm C, 64%, 73% and 18% had similar complaints, respectively. Gabapentin responders were more likely to present with verbal aggression, without delusions or gait disorders. Gabapentin doses ranged from 200–1200 mg/day (arm B, median‐ 600 mg). Arm B had the shortest median length of stay (9.55 weeks) compared to the other arms (11.7–24.85 weeks), with a significantly shorter stay in arm B than C (*p* =0.0014). In total, 143 patients received gabapentin. Of these, 13 (9.1%) had side effects leading to discontinuation. Sedation occurred in 10 (7.0%), while confusion (*n* =3), myoclonus (*n* =2), and falls (*n* =1) were also reported. Three patients experienced worsening NPS on gabapentin.

**Conclusions:**

Gabapentin was effective for treating patients with NPS in dementia. It may be an alternative drug for managing NPS in dementia, especially in cases where antipsychotic risks outweigh benefits.